# Fungal Endocarditis: A Rare Cause of Left Ventricular Outflow Obstruction

**DOI:** 10.14797/mdcvj.1241

**Published:** 2023-08-25

**Authors:** Elia El Hajj, Alexander Glaser, David Yancey, Allen Byl, Mehnaz Rahman

**Affiliations:** 1 Louisiana State University Health Sciences Center, New Orleans, Louisiana, US

**Keywords:** endocarditis, candida metapsilosis, antifungals, surgical intervention

## Abstract

A 53-year-old male presented with worsening fever, chest pain, and dyspnea during the past 2 weeks. He was hypoxic, tachycardic, and hypotensive on admission. Labs were notable for high-sensitivity troponin of 657 pg/mL and B-type natriuretic peptide of 1,648 pg/mL. Chest imaging was consistent with acute respiratory distress syndrome. Transthoracic echocardiography revealed an ejection fraction of 30% to 35% and a mobile 1.5 cm x 1.6 cm hyperechoic mass on the ventricular surface of the aortic valve (AV) with left ventricular outflow obstruction and mean pressure gradient of 38.7 mm Hg and maximum velocity of 3.64 m/s. The patient was initiated on empiric antibiotic and antifungal therapy. Cardiothoracic surgery was consulted for urgent AV repair. Blood cultures were positive for *Candida metapsilosis*, and intravenous fluconazole and micafungin were initiated. Despite aggressive and prompt medical management, the patient sustained cerebral embolic events in the middle cerebral artery territory and passed away.

## Introduction

Fungal endocarditis is a rare condition accounting for only 2% to 4% of all infective endocarditis (IE) cases.^1^ Despite improved diagnostic measures and management, fungal endocarditis remains a deadly disease with an in-hospital mortality rate as high as 54%.^2^ Fungal endocarditis primarily occurs in the setting of indwelling catheters, prosthetic valves, IV drug use, or immunosuppression.^1^ Among the causes of IE, *Candida albicans* is the most common fungal source.^3^ *Candida metapsilosis* is another form of *Candida* but is an uncommon source of IE due to its low virulence and poor adhesion to human epithelial cells.^4^ As such, infection of a native valve with *Candida metapsilosis* in an immunocompetent host lacking significant risk factors is highly unlikely.

## Case Presentation

A 53-year-old incarcerated White male with untreated hepatitis C virus (HCV) and decompensated cirrhosis presented to our institution after 2 weeks of worsening subjective fevers, progressive abdominal pain and distension, cough with hemoptysis, dyspnea, and a diffuse purpuric rash. Vital signs at presentation were notable for hypoxia requiring 6 L oxygen by nasal cannula, hypotension with systolic blood pressure in the 90s, and tachycardia.

Physical exam revealed extensive petechia and purpura along the trunk, all four extremities, and face; conjunctival pallor; abdominal distension with fluid wave; hepatosplenomegaly; 2+ pitting edema to the knees on bilateral lower extremities; and diffuse crackles bilaterally in his lungs. He became progressively more dyspneic and hypotensive with increased labored breathing, prompting emergent intubation, mechanical ventilation, vasopressor support, and admission to the intensive care unit (ICU). There was no evidence of pulmonary embolism on computed tomography; however, there were bilateral pleural effusions and multifocal ground glass opacities.

Labs revealed hemoglobin of 7.3 g/dL, platelets of 121 K, blood urea nitrogen of 32, serum creatinine of 2.17 mg/dL, aspartate transaminase of 63 U/L, alanine transaminase of 112 U/L, brain natriuretic peptide of 1,648 pg/mL, high-sensitivity troponin of 657 pg/mL, and hepatitis C viral load > 280,000. Urinalysis revealed 11-15 red blood cells and proteinuria > 30 g, with few white blood cells. Given the evidence of hematuria with acute renal injury, complement levels were checked and notable for significant hypocomplementemia (low C3, C4, and CH50). Autoimmune markers were negative for antinuclear antibodies, antineutrophil cytoplasmic antibodies, and cryoglobulins, but he did have positive rheumatoid factor (RF 81) with history of arthralgias and joint pains. Punch biopsy of his petechial rash was consistent with leukocytoclastic vasculitis, likely due to his untreated HCV. He was started on broad-spectrum empiric antibiotics (vancomycin and cefepime) due to concern for septic shock. Bronchoscopy was performed given his hemoptysis and did not show progressive blood on serial aliquots.

**Figure 1:**
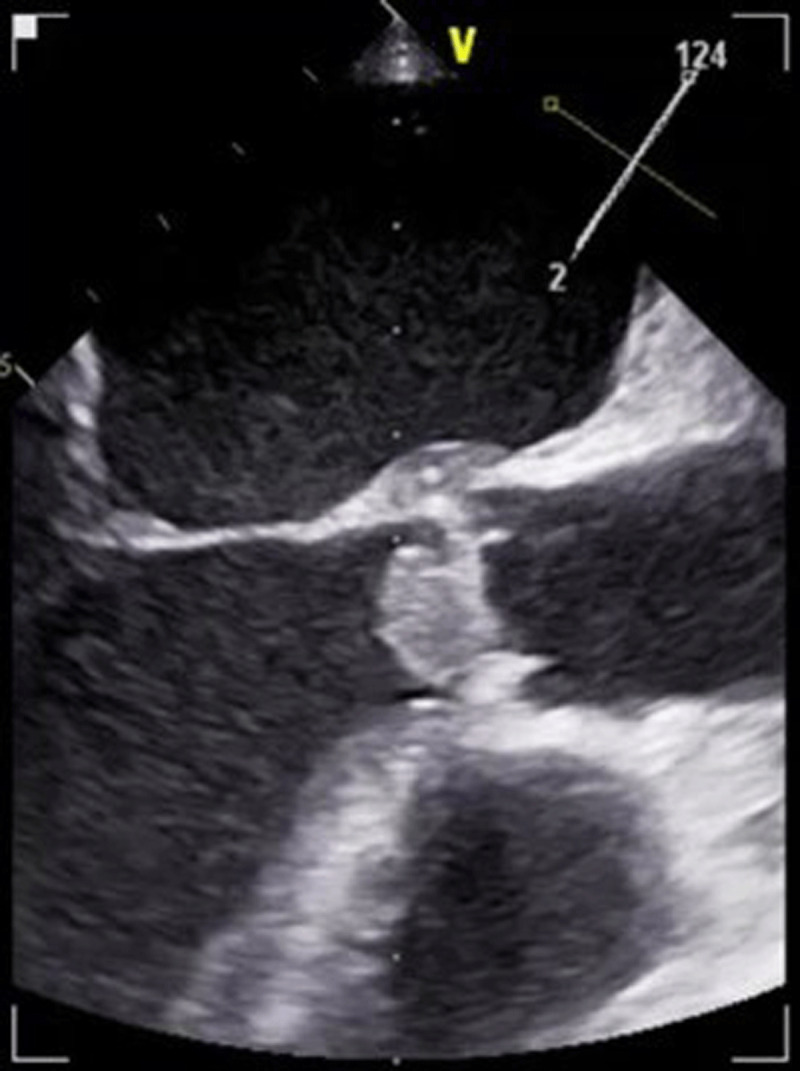
Large echogenic mass can be appreciated on the aortic valve.

**Figure 2:**
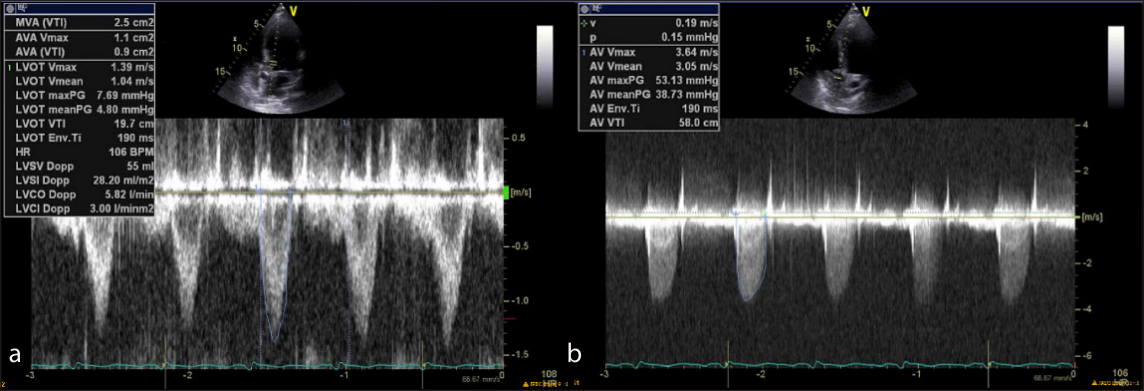
Pulse wave Doppler (2a) and continuous wave Doppler (2b) with evidence of left ventricular outflow obstruction.

Transthoracic echocardiography was performed and demonstrated global left ventricular (LV) hypokinesis, right ventricular dilation and hypokinesis, and a 1.5 cm x 1.6 cm echogenic mass on the ventricular side of the aortic valve (AV) (Figure 1). Transesophageal echocardiography demonstrated a large mobile vegetation on the AV with LV outflow obstruction (Video 1). AV mean pressure gradient was 38.7 mm Hg and the maximum velocity was 3.64 m/s (Figure 2). The calculated dimensionless index was 0.34 (Figure 2).

**Video 1:** Transesophageal echocardiography midesophageal aortic valve short axis view (left) and long axis view (right). Large mobile vegetation on the aortic valve with significant left ventricular outflow obstruction; see also at https://youtu.be/nNkrE-YsOKk.

**Figure 3:**
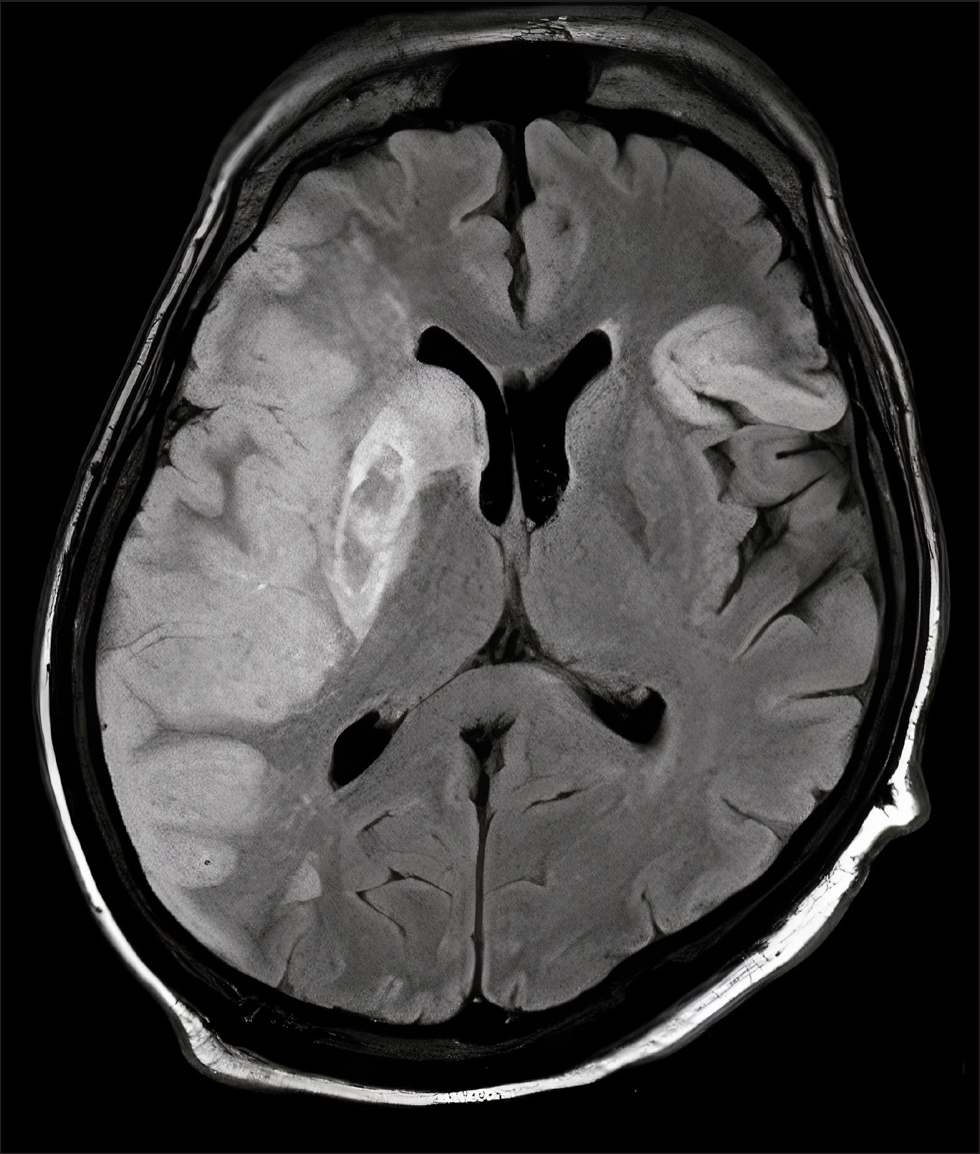
Magnetic resonance imaging of head indicating right-sided middle cerebral artery stroke.

Although blood cultures remained negative at 48 hours, he continued to have a fever with increasing pressor requirements. Fungitell ultimately returned positive (> 500) and IV liposomal amphotericin was added, pending yeast speciation. Patient’s cefepime was de-escalated to ceftriaxone and azithromycin for community-acquired pneumonia coverage. Cardiothoracic surgery was consulted to evaluate for urgent AV repair. Fungal cultures subsequently returned positive for *Candida metapsilosis* and remained positive throughout all repeat cultures, solidifying concern for septic shock secondary to native aortic valve fungal infective endocarditis. Antifungal regimen was switched to IV fluconazole and micafungin. On day 5 of his ICU stay, the patient became difficult to arouse, prompting imaging that revealed acute cerebrovascular accident (Figure 3), likely secondary to septic embolization. Given that his pressor requirements were driven by underlying sepsis with progressive multisystem organ failure, surgical intervention was deferred. Unfortunately, the patient’s clinical status continued to decline, with worsening mental status and MRI evidence of midline shift, and the patient ultimately expired.

## Decision Making

According to the American College of Cardiology/American Heart Association guidelines, early surgical intervention within 24 hours is primarily recommended in patients with IE presenting with congestive heart failure (CHF). However, the most common complication associated with fungal IE is systemic embolization followed by CHF. Despite prompt medical therapy, the risk for embolism remains high. This was evident in our patient, who sustained a large and fatal middle cerebral artery (MCA) stroke despite early identification and initiation of antimicrobial therapy. Although guidelines recommend urgent surgical intervention within 7 days, this patient population may also benefit from early intervention. However, decision making is further complicated by patients’ comorbidities and their acuity on presentation.

## Discussion

*Candida* accounts for only 1% to 2% of all cases of IE but is associated with a very high mortality rate, with one-third of patients dying during their hospitalization and another one-third dying within 1 year.^5^ Despite advances in the diagnosis and management of IE over the past 2 decades, mortality rates have not changed significantly.

The presentation of IE is highly variable, and many patients are asymptomatic on presentation. Our patient presented with progressive abdominal pain and distension, cough, fevers, and dyspnea. The presentation was initially concerning for decompensated liver cirrhosis secondary to untreated HCV; however, a diagnosis of IE was made using the modified Dukes criteria. Our patient met both major criteria, including positive blood cultures and evidence of endocardial involvement, as well as multiple minor criteria including intravenous drug use, fever, embolic phenomena, and immunologic phenomena (possible glomerulonephritis). Although the Duke criteria plays a key role in the diagnosis of IE, it offers no prognostic value, which may help guide the need for early aggressive management.

Management of IE continues to pose a challenge as physicians weigh the risks versus benefits of early surgical intervention. No randomized controlled trials have been conducted to guide current practice. Studies have shown a decreased risk of in-hospital and long-term mortality in patients undergoing an early invasive strategy versus medical management.^6^ Despite this, the timing of surgical intervention remains unclear, and guidelines are largely based on small case series and expert opinions. This decision making is further complicated by various comorbidities that significantly increase surgical risk. This complex nature is likely the reason that the European and US guidelines differ significantly regarding surgical timing.

In our case, multiple indications were present that suggested benefit for urgent surgical intervention within days. These indications include difficult organisms (ie, fungal), a very large vegetation (> 15 mm) without embolic complications, and periannular involvement. We opted for medical management due to serious comorbidities, including liver cirrhosis and septic shock, as well as the absence of heart failure symptoms or conduction abnormalities.

The most common complications associated with fungal endocarditis include systemic embolization (34%) followed by congestive heart failure.^7^ The risk for systemic embolization is highest during the first few days following antifungal therapy. Large vegetations (> 15 mm) are associated with a high risk of recurrent or new embolic events when treated with medical management alone.^8^ This may be due to poor penetration of antifungal agents when treating larger fungal masses.^4^ The majority of IE emboli involve the central nervous system (> 60%) and approximately 90% of those arise in the MCA.^9^ Unfortunately, this was the case in our patient who sustained a massive and fatal MCA stroke.

## Conclusion

Diagnosis and treatment of IE continue to pose a challenge, as evident by the poor prognosis and high mortality rate (~50%) associated with this diagnosis. This case highlights the challenges of managing high-risk patients in extremis with valvular IE. More data is needed to identify patients who may best benefit from early invasive strategy.
